# Development and validation of a measure of concrete and abstract thinking

**DOI:** 10.1371/journal.pone.0320009

**Published:** 2025-04-17

**Authors:** Hjördis Lorenz, Esther Beierl, Gabriella Tyson, Jennifer Wild

**Affiliations:** Oxford Centre for Anxiety Disorders and Trauma, The Old Rectory, University of Oxford, Oxford, United Kingdom; Hangzhou Normal University, CHINA

## Abstract

Abstract-analytical thinking, which characterizes rumination and worry, has been shown to be a risk and maintenance factor for psychological disorders, yet there are no accessible, reliable measures that can be easily administered to assess this cognitive process. Student paramedics are at elevated risk of developing mental health difficulties associated with rumination and worry due to the nature of their work. The current study describes the development and validation of the Concrete and Abstract Thinking measure (CAT) in a sample of student paramedics. The scenario-based CAT measure was systematically developed. An initial pool of scenarios was generated based on previous research and the Worry Domains Questionnaire. A total of 14 paramedics, inclusive of student paramedics, evaluated the content of the scenarios. Final items were determined based on best-fit using confirmatory factor analysis. Two-hundred student paramedics completed the CAT measure and associated measures and 96.6% completed it again for test-retest reliability. Abstract items of the CAT measure showed good internal consistency (α=.87), test-retest reliability (ICC = .88) and good factorial, construct and criterion validity. The CAT measure was significantly associated with measures of perseverative thinking (*r* = .52), rumination (*r* = .42), worry (*r* = .50), depression (*r* = .32), anxiety (*r* = .41), posttraumatic stress disorder (*r* = .23), self-efficacy (*r* = -.32) and resilience (*r* = -.30). Overall, the CAT measure showed robust psychometric properties, evidencing good validity and reliability. The CAT measure offers a user-friendly, valid, reliable and population-specific measure of concrete and abstract thinking whilst also providing a model of how abstract thinking could be assessed in a range of populations at risk of developing mental health disorders.

## Introduction

Rumination and worry are cognitive processes linked to distressing emotions [1–4]. Rumination, defined as repetitive negative thinking about past experiences, maintains low mood [e.g. 5,6] and predicts the onset and persistence of posttraumatic stress disorder (PTSD; [1,3,7], whereas worry, repetitively thinking about potential negative events, has been linked to anxiety [e.g. 8]. For populations at elevated risk of developing mental health disorders, such as students [9–11], paramedics [12–15], or student paramedics (paramedics in training), ruminative thinking has been tied to the onset and development of depression, anxiety and PTSD [7,16,17]. Research has identified abstract-analytical thinking as a core cognitive feature of rumination and worry that links to mental health problems [18–21]. It is associated with a concurrent reduction in concrete-experiential thinking [22,23]. There is a paucity of research on specific features of rumination, such as abstract thinking, that increase risk for mental ill health. One of the challenges impeding investigation is the lack of accessible, reliable and valid measures of abstract and concrete thinking that could be easily administered to at-risk populations. For paramedics, such a measure could facilitate the early identification of risk factors likely to increase psychopathology over the course of their careers. Assessing features of rumination could provide a better understanding of targets for intervention, and could be used during the course of treatment to evaluate changes in relevant cognitive processes. This paper describes the development and validation of a measure of concrete and abstract thinking (CAT) for student paramedics

### Concrete and abstract thinking

Abstract-analytical thinking is a mode of cognitive processing characterized by over-general thoughts of overall meaning as well as ‘why?’ and ‘what if?’ questions with no obvious answer. Abstract thinking often focuses on causes, meanings and consequences [24], and may include thoughts, such as “why is this always happening to me?” or “what if I never get over this?”. Concrete-experiential thinking, on the other hand, focuses on how an event is happening, on direct experience, and on means to desired ends (e.g., steps needed to achieve a goal) with ‘how?’ and ‘what?’ questions [25], such as “how can I learn from this?”, “what can I do next?”. Concrete-experiential thinking has been associated with adaptive psychological coping, including improved mood [26], better problem solving ability [27] and proactive behavior [28]. Concrete thinking may also relate to general resilience and self-efficacy, although this is yet to be tested.

Studies have highlighted the association between anxiety-related worry and abstract thinking. Worries described in a clinical sample with generalized anxiety disorder (GAD) were more abstract than those of a non-clinical control group, and became significantly more concrete after cognitive behavioral therapy [19]. Abstract thinking has also been explored in relation to depressive rumination [27,29], whilst concrete thinking has been the focus of some forms of successful treatment for depression and anxiety [30,31]. Initial research has linked abstract thinking to PTSD where specific features of rumination, including ‘why’ and ‘what if’ questions appear to predict PTSD beyond trauma history [32] and beyond rumination alone [18]. Abstract processing during an analogue trauma film increased rumination [33] and abstract thinking after a trauma analogue film led to a significantly longer maintenance of negative mood and arousal, compared to concrete thinking or distraction [34]. Overall, research on abstract thinking is still in its infancy and is held back by a lack of user-friendly, psychometrically sound measures, as detailed below.

### Existing measures of concrete and abstract thinking

The majority of studies that assess the abstractness or concreteness of specific thoughts have administered the *Problem Elaboration Questionnaire* [PEQ, 19]. Although the PEQ is validated and widely used, it has a number of limitations. First, the PEQ instructs participants to elaborate on two problems they are currently worried about. This wording may directly encourage participants to worry and may thereby induce a more abstract focus, thus biasing responses towards abstract content. Second, since no word or time limit is given, elaborations may vary greatly in length which again may influence scoring. One-word or short answers may bias towards abstract scoring when they could simply be a representation of writing preferences. Third, applying a 5-point scale with only two anchors at the extreme ends heightens subjectivity in scoring, as individuals could differ in their understanding of scale-points 2, 3, and 4 when these are not labelled. Fourth, the definitions of concrete and abstract thinking used in the PEQ differ from definitions adopted in more current research [e.g., 20], which may limit the comparability of results. Finally, the questionnaire can be lengthy to administer and score, as individuals need to type out responses instead of ticking multiple choice options, and scoring needs to be done by hand instead of receiving an automatic sum score. As length can add to questionnaire burden for users, and limit use for busy practitioners, this is a limitation to consider.

A few studies have assessed concrete and abstract thinking using other measures, however, with considerable limitations related to the use of different definitions of abstract thinking [35], unstable factorial validity [36] or measures not validated or published in English [22,37]. Some studies asked participants to self-identify abstract or concrete thoughts [38] with items such as “my dwelling is usually very abstract” [39]. These two measures put the responsibility of understanding and identifying the concept of abstract thinking solely on participants which may increase the risk of inaccuracies and social desirability bias [40].

In summary, existing measures of abstract and concrete thinking demonstrate a number of limitations including a biased wording towards inducing worry, scoring impacted by participants’ elaboration preference, potential subjectivity in scoring, varying definitions of abstract thinking, lengthy completion time, unpublished and unvalidated measures or reliance on participants to self-identify abstract thinking.

### Development of the concrete and abstract thinking measure (CAT)

The *Concrete and Abstract Thinking measure (CAT)* was developed as a scenario-based measure that includes an assessment of abstract and concrete thoughts associated with each scenario. Scenarios were chosen to address the PEQ’s limitations surrounding variations in the length of elaboration for each question, subjectivity in scoring, and differences in completion time. An initial pool of scenarios was generated, drawing on previous research of the Worry Domains Questionnaire [41,42] and examples of concrete and abstract thinking provided in published literature [20,43]. The CAT scenarios differed from previous research [41,42] in that they did not ask participants to describe a worry scenario but rather presented participants with a difficult situation likely to occur at university (e.g., submitting an essay late) or in paramedic work (e.g., trying to intervene with a patient in cardiac arrest), which *could* trigger worry or rumination. These initial scenarios were evaluated by 16 student and qualified paramedics and rated on a scale from 0–100% on how realistic they were and how likely they would be to cause individuals to worry or ruminate. If scenarios were rated as less than 60% realistic, they were replaced with new scenarios proposed by the paramedics and re-evaluated.

Four abstract and four concrete thought responses (items) were provided for each scenario. This was designed to address previous limitations associated with social desirability bias and participants’ conceptual understanding of abstract and concrete thinking. That is, unlike previous research, there was no expectation for individuals to identify whether or not their thoughts were concrete or abstract. The concrete and abstract thoughts for each scenario were developed in collaboration with a Clinical Psychologist, a User Advisory Group, consisting of four paramedics including student paramedics, and were based on existing research. Each scenario has 8 thought responses, four of them abstract, four of them concrete. Participants are instructed to indicate (yes/no) whether they would experience each response if they were in the scenario. The final score is calculated as the overall *ratio* of abstract to concrete thoughts for each scenario by dividing the number of abstract responses endorsed by the number of concrete responses endorsed, to give an indication of whether participants thought *more* concretely or *more* abstractly.

As part of the validation, 16 scenarios were developed (8 related to paramedic work and 8 to university work). Confirmatory factor analysis was applied to determine the scenarios and to shorten the questionnaire. Confirmatory factor analysis of the original 16 scenarios led to a two-factor model. Four scenarios were selected based on factorial validity, construct and criterion validity, as well as theoretical and clinical considerations, to create the final CAT measure, resulting in two scenarios related to paramedic work and two related to university work. See S1 Measure for the final CAT items.

The aims of the present study were to (1) assess the psychometric properties of the CAT measure including construct and criterion validity and to (2) investigate the relationship between the CAT measure and related concepts of worry, rumination and repetitive negative thinking, as well as measures of psychopathology (generalized anxiety disorder, depression, PTSD), self-efficacy and resilience. It was hypothesized that abstract thinking, as measured by the CAT measure, would be positively associated with measures of abstract thinking, repetitive negative thinking, worry, rumination, generalized anxiety disorder (GAD), depression and PTSD and negatively correlated with self-efficacy and resilience.

## Method

### Participants

All participants were British student paramedics completing a 3-year Bachelor in Paramedic Science. Recommendations for factor analyses [44] suggest recruiting a sample size that includes 10 times as many participants as measure items. For 16 original scenarios, and adjusting for a potential 20% rate of attrition, we aimed to recruit 200 participants. The final sample included N = 205 student paramedics from 15 universities with an age range of 18–54 (*M* = 24.91, *SD* = 6.77). The majority were female (62.0%; 38% male) and White British (92.19%). Other ethnicities included 1.46% White Irish, 1.95% Eastern European, 1.95% another White Background, 0.49% Caribbean, 0.98% White and Asian, and 0.98% White and Black Caribbean. The socially constructed groupings of age, gender, and ethnicity were categorized in line with standardized recommendations by the Medical Sciences Inter-Divisional Research Ethics Committee at the University of Oxford. Age allowed for an open response of any number, gender included the options “male”, “female”, “other” and “prefer not to say,” and ethnicity provided a selection of 18 options as well as an open response option related to “other background.” The authors thereby had access to identifying information of participants during data collection. All identifiable information was stored separately to outcome data. All outcome data was anonymized immediately after data collection for analysis. A sample of 205 participants completed the set of questionnaires at time point 1 and 198 participants (96.6%) completed the questionnaires again at time point 2, two weeks later, in line with recommendations for health measurement scales [45]. The seven participants who dropped out from the first to the second time point could not be reached.

### Instruments

#### Concrete and abstract thinking measure (CAT).

The CAT measure, as described above, was used and is the focus of this study. The current study calculated the overall *ratio* of abstract to concrete thoughts for each scenario by dividing the number of abstract responses endorsed by the number of endorsed concrete responses, to give an indication of whether participants thought *more* concretely or *more* abstractly. This will be referred to as the ‘abstract ratio’ with higher scores indicating greater abstractness.

#### Additional measure of abstract thinking.

*The Problem Elaboration Questionnaire* [19], described above, was used. The PEQ instructs participants to elaborate on two problems they are ‘currently worried about’ as well as on ‘three potential negative consequences’ for each problem. These elaborations are scored for concreteness using Stöber’s 5-point concreteness rating scale [46], with scores from 1–5 for each problem/consequence. Abstract thinking is defined as ‘indistinct, cross-situational, equivocal, unclear, aggregated’ and concrete thinking as ‘distinct, situationally specific, unequivocal, clear, singular.’ A total concreteness score for major worries/problems was calculated. In the present sample, a random 10% of the PEQ was scored by a second, independent rater (GT). This showed good inter-rater reliability: problem elaboration ICC = .80, *p* < .001, 95% CI [.49,.92] and consequence elaboration ICC = .81 *p* < .001, 95% CI [.53,.93].

#### Repetitive negative thinking.

The *Perseverative Thinking Questionnaire* [PTQ, 47] is a 15-item measure of repetitive thinking independent of disorder, covering worry and rumination. Items are rated on a scale from 0 =  *never* to 4 =  *almost always,* leading to a range of scores from 0–60. The PTQ has high internal consistency α=.93-.95, acceptable test-retest reliability *r* = .69-.75 [47], and good predictive validity for symptom levels of anxiety and depression [48]. For the present sample, Cronbach’s alpha was α=.96.

#### Worry.

The *Penn State Worry Questionnaire* [PSWQ, 49] is a 16-item self-report questionnaire to assess worry which demonstrates excellent internal consistency (α=.93) and test-retest reliability *r* = .92. Questions are scored from 1–5 with 1 =  *not at all typical of me* and 5 *=  very typical of me*, with total scores ranging from 16–80. For the present sample, Cronbach’s alpha was α=.92.

#### Rumination.

The *Ruminative Response Scale* [RRS, 50] is divided into two subscales: brooding and reflective pondering. In the current study, only the 5-item brooding subscale was used which had adequate internal consistency, α=.77, and test-retest reliability, *r* = .62 [50]. Scores are rated from 1–4 with 1 = *almost never* to 4 = *almost always*, leading to a range of scores from 5–20. For the present sample, Cronbach’s alpha was α=.78.

#### Anxiety.

The *Generalized Anxiety Disorder Scale* [GAD-7, 51] is a 7-item questionnaire assessing the frequency of generalized anxiety symptoms over the previous week. Scores range from 0 =  *not at all to* 3 =  *nearly every day*, with a range from 0–21. The GAD-7 showed excellent internal consistency (α=.92) and good test-retest reliability (ICC = .83). For the present sample, Cronbach’s alpha was α=.91.

#### Depression.

The *Patient Health Questionnaire* [PHQ-9, 52] is a 9-item self-report questionnaire based on the DSM-IV [53] criteria for depression and assesses symptoms of low mood over the previous two weeks. Scores range from 0 =  *not at all* to 3 =  *nearly every day*, with a range of total scores from 0–27. Kroenke and team reported good internal reliability (α=.89) and test-retest reliability with a kappa of .84 after 48 hours. For the present sample, Cronbach’s alpha was α=.85.

#### PTSD.

The *PTSD Checklist for DSM-5* [PCL-5, 54] is a 20-item measure of PTSD symptoms directly corresponding to the DSM-5 PTSD criteria [55]. Symptoms are rated on a scale from 0 =  *not at all* to 4 =  *extremely*, with a range of 0–80. Psychometric evaluation of the PCL-5 with university students exposed to trauma showed excellent internal consistency (α=.94), and test-retest reliability (*r* = .82) [54]. For the present sample, Cronbach’s alpha was α=.95.

#### Self-efficacy.

To better understand abstract thinking related to mental health and wellbeing, measures of resilience were included. The *General Self-Efficacy Scale* [56] is a 10-item scale that assesses the capacity to be self-reliant and effective in problem-solving. Items are rated on a *1 = not at all true* to *4 =  exactly true* scale with total scores ranging from 10 to 40. The GSE demonstrates good internal reliability (Cronbach’s alphas α=.76-.90). It has been shown to correlate with measures of optimism and work satisfaction, and to negatively correlate with depression, stress, health complaints, burnout, and anxiety. For the present sample, Cronbach’s alpha was α=.84.Resilience. The Resilience Scale [57] is a 25-item measure of resilience with good internal consistency (α=.91) in an elderly, non-clinical sample. Items range from 0 =  *not true at all* to 4 =  *true nearly all the time*, with a total score range from 0–100. Test-retest reliability was assessed in a study of pregnant and postpartum women [58] as cited by [57] and ranged from *r* = .67 to.84. For the present sample, Cronbach’s alpha was excellent, α=.94. The *Connor-Davidson Resilience Scale* [CD-RISC, 59] is a 25-item measure of resilience with good reliability and validity. Internal consistency was high with Cronbach’s α=.89 and test-retest reliability, ICC = .87, in a clinical sample with PTSD and GAD. For the present sample, Cronbach’s alpha was α=.91.

### Procedure

The Medical Sciences Inter-Divisional Research Ethics Committee at the University of Oxford granted approval for the study (R57540/RE002). Participants were recruited between September 2018 and January 2019. A criterion sampling method was applied which included any British student paramedics. This was deemed suitable as courses are highly regulated in their content by the UK’s National Health Service (NHS) and therefore offer similar training. Invitations to participate were emailed to 11 paramedic university courses. Some universities passed on the invitation to 4 further partnering UK paramedic courses (snowball sampling method). This led to student paramedics from 15 universities consenting to participate based on voluntary interest without a pre-determined number of participants per university. The only inclusion criteria was current enrollment in a UK paramedic course. Written informed consent was given by all participants. Participants completed an approximately 30-minute online set of questionnaires using Qualtrics software at time point 1 and two weeks later at time point 2. Upon completion of the second set of questionnaires, participants received a £20 Amazon voucher. All data were collected in 2018–19 before the Covid-19 pandemic.

### Analyses

Analyses were conducted using SPSS [Version 25, 60], the R ‘lavaan’ package [Version: 0.6–3, [61] and Rstudio [Version 1.1.463, 62]. Data are available upon individual request.

#### Preliminary analyses: factorial validity.

To help establish factorial validity of the four scenarios to be used in the CAT measure, confirmatory factor analyses (CFAs) were conducted for each of the 16 proposed scenarios. Sixteen CFAs were conducted, each with 8 items (4 abstract, 4 concrete). A weighted least squares means and variance adjusted (WLSMV) estimation was applied since the CAT items were binary [63,64]. As the chi-square statistic increases with sample size and leads to rejection of the hypothesized model, even with good fit [65], additional fit indices were examined: the Comparative Fit Index (CFI), the Root Mean Square Error of Approximation (RMSEA), and the Standardized Root Mean Square Residual (SRMR). Variances of the latent variables were set to one.

#### Internal consistency.

Internal consistency was calculated for all abstract items and all concrete items using Cronbach’s alpha [44].

#### Test-retest reliability.

Using the 198 participants (96.6% of total sample) who completed the CAT measure a second time, two weeks after initial completion, the intraclass correlation coefficient (ICC) was calculated to assess test-retest reliability.

#### Construct validity.

To examine construct validity, specifically convergent validity, correlations were calculated to assess the relationship between the CAT measure’s abstract ratio and measures that asses a similar or related construct. Convergent validity is understood as the extent to which two measures that theoretically should be related, are in fact related. This included the PEQ (problem elaboration and consequence elaboration) as well as repetitive negative thinking (using the PTQ), worry (using the PSWQ) and rumination (using the RRS), all of which have previously been shown to be strongly correlated with reduced concreteness.

#### Criterion validity.

To assess broader constructs which have been shown to correlate with rumination or worry, correlations were calculated between the CAT measure’s abstract ratio and measures of GAD, depression and PTSD. Constructs that were expected to have a negative correlation with the CAT measure’s abstract ratio were also assessed, specifically measures of self-efficacy and resilience.

### Results

#### Reliability

##### Internal consistency.

Utilizing the entire sample (N = 205) the CAT measure (raw scores of the 4 final scenarios) demonstrated good internal consistency of abstract items (16 items), Cronbach’s α=.87 and concrete items (16 items), Cronbach’s α=.85.

##### Test-retest reliability.

The CAT measure demonstrated good test-retest reliability as measured by intraclass correlation coefficients (ICC) between both time points. Sum scores for all abstract items were correlated with the sum scores two weeks later ICC = .88, p < .001, 95% CI [.84,.91] and the same process was applied to the concrete items, ICC = .85, p < .001, 95% CI [.80,.89]. There was also adequate test-retest reliability for the abstract ratio of the CAT measure, ICC = .75, p < .001, 95% CI [.69,.81].

### Validity

#### Confirmatory factor analysis.

Table 1 shows the fit indices of the confirmatory factor analyses for each scenario. Based on factor analyses, the four scenarios with the best fit (based on the CFI, RMSEA and SRMR, parameter estimation, factor loading and content of the scenarios) were selected for inclusion in the final CAT measure. The factor loading of the items of this version ranged from.33 to.95. Table 2 shows the factor loadings of each item of the CAT measure (see S2 Table for an extended table that includes factor loadings for all 16 original scenarios). The concrete and abstract factors were negatively correlated with each other ranging from *r* = -.33 to *r* = -.39 although one scenario (scenario 4) had a positive correlation between the factors of *r* = .51.

**Table 1:** Results of scenario-wise confirmatory factor analyses (CFAs) for the CAT measure.

Scenario	χ^2^	CFI	RMSEA	SRMR
1	χ^2^(19) = 31.77, *p* = .033	.97	.06	.10
2	χ^2^(19) = 31.33, *p* = .037	.96	.06	.09
3	χ^2^(19) = 23.80, *p* = .204	.99	.03	.07
4	χ^2^(19) = 12.84, *p* = .847	1.00	.00	.06

*Note.* χ^2^ =  robust chi-square statistic; CFI =  Comparative Fit Index; RMSEA =  Root Mean Square Error of Approximation; SRMR =  Standardized Root Mean Square Residual.

**Table 2:** Factor loadings of final CAT items.

Scenario	Abstract item nr	Abstract factor loading	*p*-value	Concrete item nr	Concrete factor loading	*p*-value
Scenario 1	1	.707	<.001	2	.508	<.001
	3	.939	<.001	5	.795	<.001
	4	.840	<.001	6	.654	<.001
	7	.802	<.001	8	.453	<.001
Scenario 2	2	.730	<.001	1	.329	.004
	4	.748	<.001	3	.590	<.001
	5	.739	<.001	6	.677	<.001
	7	.877	<.001	8	.844	<.001
Scenario 3	1	.766	<.001	2	.818	<.001
	3	.890	<.001	5	.881	<.001
	4	.893	<.001	6	.496	<.001
	7	.789	<.001	8	.758	<.001
Scenario 4	1	.330	.005	3	.555	<.001
	2	.573	<.001	4	.889	<.001
	5	.662	<.001	6	.598	<.001
	7	.559	<.001	8	.472	<.001

**Fig 1:**
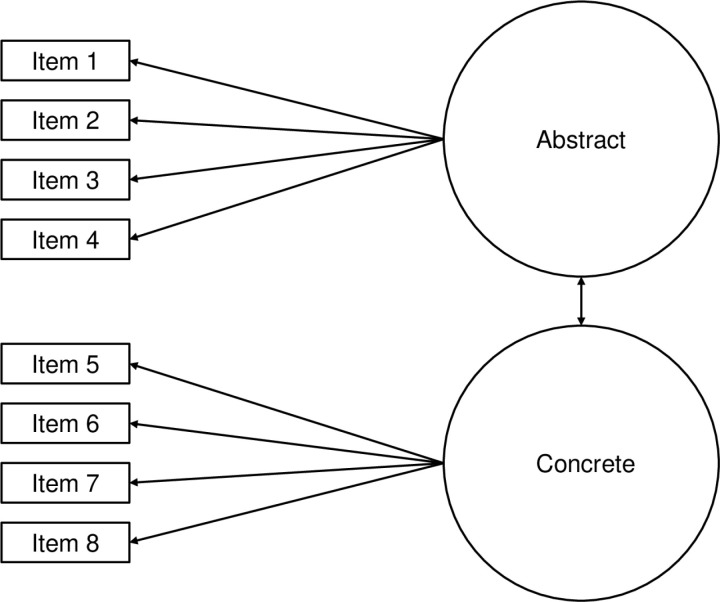
Model of concrete and abstract factor loadings. *Note.* Model of concrete and abstract factors loading onto items of the concrete and abstract thinking (CAT) measure. The lines with one arrow indicate factor loadings. The lines with two arrows indicate a correlation.

Fig 1 represents a two-factor model, as hypothesized for each confirmatory factor analysis, where one factor (abstract thinking) loads onto 4 items (thought responses) and the second factor (concrete thinking) loads onto 4 different items.

### Construct validity

All correlations were conducted with the abstract ratio. Concrete ratios were not described as they would have simply resulted in a correlation in the opposite direction. Table 3 shows an overview of all correlations.

**Table 3:** Correlations between the final CAT measure’s abstract ratio and other measures (N = 205).

Measure	*r*	*p*
PTQ	.52	<.001
PSWQ	.50	<.001
RSS	.42	<.001
PEQ problem elaboration	-.06	.24
PEQ consequence elaboration	.03	.38
GAD-7	.41	<.001
PHQ-9	.32	<.001
PCL-5	.23	=.001
GSE	-.32	<.001
Resilience Scale	-.30	<.001
CD-RISC	-.40	<.001

*Note.* PTQ=Perseverative Thinking Questionnaire; PSWQ=Penn State Worry Questionnaire; RSS=Rumination Response Scale-Brooding Subscale; PEQ=Problem Elaboration Questionnaire; PCL-5 = PTSD Checklist for DSM-5; PHQ-9 = Patient Health Questionnaire; GAD-7 = Generalized Anxiety Disorder Scale; GSE=General Self-efficacy Scale; CD-RISC=Connor-Davidson Resilience Scale.

## Discussion

The current study describes the development and psychometric evaluation of the new Concrete and Abstract Thinking measure (CAT). It aimed to address shortcomings of existing measures and offer a focus on student paramedics, a population at higher risk for mental health problems compared to the general population [9–15,66]. Overall, the CAT measure demonstrated good validity and reliability.

### Measure development

The CAT scenarios and abstract/concrete thoughts (items) were developed in collaboration with paramedics, student paramedics and a clinical psychologist specializing in PTSD. The CAT scenarios demonstrated good factorial validity based on fit, parameter estimation, factor loading and scenario content. The content of the scenarios was balanced to equally represent potentially stressful scenarios related to university and paramedic training. The above experts, after reviewing the scenarios, concluded the scenarios represented good face validity. Content validity was not evaluated quantitively but was based on 1) existing theoretical knowledge, 2) existing measures in the field, 3) judgement of experts by experience (paramedics and student paramedics) and experts by training (clinical researchers). One scenario (‘Your computer crashed with your essay on it and you don’t have a back-up’) had a positive correlation between the abstract and concrete factors. This scenario is likely common among university students and may elicit abstract thoughts in the immediate aftermath followed quickly by concrete thoughts given that essays are linked to deadlines, which could lead to more active problem-solving.

### Primary analyses

Consistent with the hypotheses, the CAT measure exhibited good construct and criterion validity relative to measures of related concepts. As hypothesized, abstract thinking was most strongly correlated with repetitive negative thinking followed by worry and rumination, cognitive processes thought to be characterized by abstract thinking [1,19,23,67,68]. Abstract thinking correlated moderately with depression and anxiety, disorders that feature rumination or worry in their symptomology [19,29,67]. Abstract thinking showed a weak correlation with PTSD, where rumination or abstract thinking have been shown to predict PTSD symptoms [7,32]. As hypothesized, abstract thinking correlated negatively with measures of self-efficacy and resilience. To the authors’ knowledge, this is the first study that has shown a relationship between these concepts, although correlations with other measures of adaptive coping such as problem solving have previously been reported [27].

In the current sample, the alternative measure of abstract thinking, the PEQ, failed to correlate significantly with measures of repetitive negative thinking, rumination, worry, depression, GAD, self-efficacy and resilience as well as the CAT measure. This is inconsistent with previous literature where the PEQ correlated significantly with concepts of repetitive negative thinking, rumination and worry, depression and GAD [19,27]. However, it is consistent with research by Ehring, Frank and Ehlers [1]. In their study of abstract thinking and rumination following traumatic road traffic accidents, the trauma-focused PEQ failed to correlate with rumination. While Stöber and Borkovec’s PEQ asks about *any* worries the participant has, the trauma-focused PEQ used by Ehring, Frank and Ehlers [1] and the CAT measure refer to *specific* scenarios or traumatic events. Ehring and colleagues suggest that focusing on worries related to a specific scenario could lead to fewer abstract thoughts than focusing on *any* worries and could facilitate problem-solving since the scenarios may be problems the participants have experience of resolving, whereas open-ended worries might include situations where participants have limited experience to draw on to problem-solve. Additionally, the limited relationship between the CAT measure and the PEQ may be related to differing definitions of concrete and abstract thinking. According to Stöber and Borkovec [19], if thoughts are ‘cross-situational and aggregated,’, they are always scored as abstract although they could be indicative of concrete thinking. For example, according to Stöber and Borkovec’s definitions, the sentence “ensuring I pass assignments and meet clinical practice milestones”, would be scored as abstract because multiple assignments and milestones meet their definition of ‘cross situational and aggregated.’ However, this example would be scored as concrete according to the definition employed in the CAT measure because it focuses on the specific steps (passing assignments) needed to achieve a goal (meeting milestones).

Our study has limitations worth noting. It is of course possible that the CAT measure did not measure the constructs of abstract and concrete thinking as intended but some different factor that correlates highly with repetitive negative thinking. However, this would seem unlikely since the CAT measure demonstrated a moderately strong relationship with the PTQ and the RRS, which measure rumination of which abstract thinking is a core feature. Other correlations, although statistically significant, were moderate in strength, meaning conclusions should be drawn with caution. However, given our sample was non-clinical, it is unsurprising that correlations between the CAT measure and GAD, depression, and PTSD symptom severity were moderate. As a next step, it would be valuable to evaluate the CAT measure in a clinical sample of student paramedics. In terms of construct validity, the CAT measure could only be compared to the PEQ, a measure with significant limitations, since there were no other validated measures of abstract thinking that could be used. This in and of itself underscores the necessity for a new, valid measure of abstract thinking. Although the use of scenarios in the CAT measure provide a good alternative to requiring individuals to self-identify abstract thinking or elaborating on problems in an open-ended response format, there are limitations to this approach. Some individuals may struggle to imagine themselves in situations while others may be prone to social desirability bias in their responses. This is an issue common to most measures. However, a strength of the CAT measure is that items are not clearly ‘right’ or ‘wrong’ and participants were not made aware that their abstract/concrete thinking was being assessed, which is the case in other measures [e.g., 39]. Content validity was not evaluated quantitatively, which may pose a limitation. Instead, it was based on theoretical knowledge, existing measures, and expert judgement. During the measure development phase, scenarios were carefully evaluated by a pilot sample for how realistic they were and how likely they would be to cause individuals to worry or ruminate. It could have been helpful to evaluate how realistic the scenarios appeared to participants in the main validation study. This might have offered insights into potential correlates between how realistic individuals found the scenarios or how vividly they were able to imagine them, and their CAT measure scores. Future research could further explore individual and overall ratings of the scenarios. Since participants only had the option of endorsing or choosing not to endorse an item, they could not select a hierarchy of thoughts, the degree to which they agreed with an item (i.e., very much, moderately), or indicate the likely frequency of the item (i.e., never, sometimes, always) on a Likert scale, which could have provided more nuanced responses. Whilst the current scoring is binary rather than continuous, it does provide a ratio of abstract to concrete thinking and a simple method for completion and scoring, which is advantageous over the PEQ. A further limitation is that a separate sample was not recruited to assess factorial validity, independently of the current sample. Due to the required sample size, the current sample was not split to create such a second sample. Overall, the study could have benefited from a larger sample for the testing of a new measure to allow for further comparisons and second factorial validation. However, the current sample was in line with recommendations on minimum sample sizes needed for factor analyses [44] and did demonstrate predictive power in a subsequent study of PTSD in student paramedics [32].

Collecting self-reported data online allowed for highly efficient data collection across wide geographical regions. However, self-report measures can be affected by the participant’s self-awareness and social desirability bias. Despite this limitation, the approach of self-reported online measures for the development and evaluation of a new self-report measure is widely used and considered valid [e.g., 47].

### Broader implications

A recent study [32] assessed the potential relationship between abstract thinking assessed with the CAT measure and the subsequent development of PTSD symptoms in a sample of 89 student paramedics. Abstract thinking at assessment predicted PTSD symptoms at 6-month follow-up over and above what could be predicted from initial symptom levels. Abstract thinking as assessed by the CAT measure was moderately related to rumination in response to stressful memories, PTSD symptoms, anxiety and depression at 6-month follow-up. This study demonstrates the extent to which the CAT measure could be used as a measure for predicting the development of psychopathology and offers initial support for abstract thinking as a risk factor in the development of PTSD symptoms in student paramedics.

For university paramedic programs, the CAT measure could provide a safe, user friendly measure of mental health risk that is quick to self-administer and that is stigma-free. The scenarios of the CAT measure could be modified and evaluated for a range of high risk populations, such as students in rescue work, medical students, student nurses or students training in a range of healthcare professions. Whilst there are limits to generalizability, it is possible that the format of the CAT measure would be relevant for such roles. During the Covid-19 pandemic, it has become clear that assessing potential risk factors for mental ill health in emergency and healthcare workers is paramount for guiding the delivery of preventative and early interventions for common mental health problems.

## Conclusion

The CAT measure provides a user-friendly, valid, reliable, and population-specific measure of concrete and abstract thinking that advances current methods of assessing cognitive features of rumination. The CAT measure demonstrates value in the assessment of abstract thinking as a risk factor for mental ill health and may offer guidance in the delivery of interventions aimed to prevent or treat common mental health problems for students in high risk occupations.

## Supporting information

S1 Measure The Concrete Abstract Thinking measure (CAT). (PDF)

S2 Table Factor Loadings of Original 16 CAT Scenarios. (PDF)
