# Supplementary material for: Development and validation of a measure of concrete and abstract thinking
Source: PLoS One. 2025 Apr 17;20(4):e0320009. doi: 10.1371/journal.pone.0320009 (PMC12005567; doi:10.1371/journal.pone.0320009)
Supplement: S1 Measure — (PDF) [file pone.0320009.s001.pdf]

Supporting Information 1

The Concrete Abstract Thinking Measure

Paramedic Scenarios:
Scenario 1: You are the first to arrive on the scene of an accident. A young man is lying on the ground. You try to resuscitate him.

1. What if I can't help him? (abstract)
2. What's the first thing I need to do? (concrete)
3. What if I forget the procedure? (abstract)
4. What if my supervisor thinks I'm doing a bad job? (abstract)
5. How can I help him? (concrete)
6. What have I learned that I can apply in this situation? (concrete)
7. What if I'm never competent with this procedure? (abstract)
8. How can I get help if this doesn't go well? (concrete)

Scenario 2: You see a patient and recognize symptoms that you learned about in class but can't remember what you have to do next.

1. Who can I ask for help? (concrete)
2. What if I mess up completely? (abstract)
3. What do know about these symptoms? (concrete)
4. Why do I struggle to remember the important stuff? (abstract)
5. What if I never learn it properly? (abstract)
6. What can I do to make the patient feel comfortable? (concrete)
7. Does this mean I'm not cut out to be a paramedic? (abstract)
8. What have I learned that I can apply in this situation? (concrete)

University scenarios:
Scenario 3: You received a bad mark for an essay.

1. What if I fail this course? (abstract)
2. How can I use the overall feedback to improve? (concrete)
3. What is wrong with me? (abstract)
4. Why can't I do anything right? (abstract)
5. What resources could help me? (concrete)
6. Who can I ask for help? (concrete)
7. What if my tutors think I'm not good enough? (abstract)
8. How can I better prepare next time? (concrete)

Scenario 4: Your computer crashed with your essay on it and you don't have a back-up.

1. Why me, why now? (abstract)
2. Why didn't I prepare for this? (abstract)
3. What steps can I take to recover it? (concrete)
4. Who or where can I go for help? (concrete)
5. What if I fail this essay? (abstract)
6. If I can't recover my essay, what can I do now to make progress? (concrete)
7. Why didn't I backup? (abstract)
8. What do I still have or remember about my essay? (concrete)
