# Supplementary material for: Development and validation of a measure of concrete and abstract thinking
Source: PLoS One. 2025 Apr 17;20(4):e0320009. doi: 10.1371/journal.pone.0320009 (PMC12005567; doi:10.1371/journal.pone.0320009)
Supplement: S2 Table — (PDF) [file pone.0320009.s002.pdf]

Supporting Information 2

Table 1 shows the factor loadings for all original 16 CAT scenarios evaluated in the confirmatory factor analysis (CFA).

Table 1
Factor Loadings of Original 16 CAT Scenarios

**Version_Scenario**	**Abstract item nr**	**Abstract factor loading**	***p*-value**	**Concrete item nr**	**Concrete factor loading**	***p*-value**
1_1	1	.448	<.001	2	.753	<.001
	3	.868	<.001	5	.419	.004
	4	.717	<.001	6	.251	.060
	7	.923	<.001	8	.173	.210
1_2	1	.703	<.001	3	.578	<.001
	2	.658	<.001	4	.675	<.001
	5	.796	<.001	7	.534	<.001
	6	.743	<.001	9	.679	<.001
	8	.588	<.001	10	.643	<.001
1_3	2	.722	<.001	1	1.295	.063
	4	.724	<.001	3	.107	.328
	5	.722	<.001	6	.094	.379
	7	.857	<.001	8	.253	.109
1_4 (Became scenario 1 in final CAT)	1	.707	<.001	2	.508	<.001
	3	.939	<.001	5	.795	<.001
	4	.840	<.001	6	.654	<.001
	7	.802	<.001	8	.453	<.001
2_1	1	.204	.103	2	.521	<.001
	3	1.034	<.001	4	.366	.006
	6	.477	<.001	5	.751	<.001
	7	.442	<.001	8	.108	.392
2_2	1	.527	<.001	2	.362	.001
	6	.713	<.001	3	.700	<.001
	8	.704	<.001	7	.280	.012
	4	.787	<.001	5	.017	.0873
2_3 (Became scenario 2 in final CAT)	2	.730	<.001	1	.329	.004
	4	.748	<.001	3	.590	<.001
	5	.739	<.001	6	.677	<.001
	7	.877	<.001	8	.844	<.001
2_4	1	.606	<.001	2	.819	<.001
	3	.961	<.001	5	.507	<.001
	4	.859	<.001	6	.376	.005
	7	.892	<.001	8	.507	<.001
	1	.766	<.001	2	.818	<.001

3_1 (Became scenario 3 in final CAT)	3	.890	<.001	5	.881	<.001
	4	.893	<.001	6	.496	<.001
	7	.789	<.001	8	.758	<.001
3_2 (Became scenario 4 in final CAT)	1	.330	.005	3	.555	<.001
	2	.573	<.001	4	.889	<.001
	5	.662	<.001	6	.598	<.001
	7	.559	<.001	8	.472	<.001
3_3	2	-.260	.025	1	.120	.340
	4	-.212	.048	3	.796	<.001
	5	-.650	<.001	6	.728	<.001
	8	-.208	.098	7	.416	<.001
3_4	1	-.518	<.001	2	.812	<.001
	3	-.014	.901	5	.738	<.001
	4	-.923	<.001	6	-.311	.003
	7	-.082	.576	8	.527	<.001
4_1	1	-.780	<.001	2	.637	<.001
	3	-.095	.425	4	.395	.001
	6	-.713	. <.001	5	.474	<.001
	7	-.266	.019	8	.362	.002
4_2	1	.311	.003	2	.924	<.001
	4	.458	<.001	3	-.209	.050
	6	.185	.092	5	.600	<.001
	7	-1.029	<.001	8	.862	<.001
4_3	2	.372	.002	1	.133	.252
	4	.841	<.001	3	.845	<.001
	5	.405	.001	6	.844	<.001
	7	.735	<.001	8	.639	<.001
4_4	1	.381	<.001	2	.426	<.001
	3	.156	.166	5	.462	<.001
	4	-.725	<.001	6	-.409	.002
	7	.461	<.001	8	.731	<.001
